# Gossypol Acetic Acid Attenuates Cardiac Ischemia/Reperfusion Injury in Rats via an Antiferroptotic Mechanism

**DOI:** 10.3390/biom11111667

**Published:** 2021-11-10

**Authors:** Jian-Hong Lin, Kun-Ta Yang, Pei-Ching Ting, Yu-Po Luo, Ding-Jyun Lin, Yi-Shun Wang, Jui-Chih Chang

**Affiliations:** 1 Division of Experimental Surgery, Department of Surgery, Hualien Tzu Chi Hospital, Buddhist Tzu Chi Medical Foundation, No. 707, Sec. 3, Zhongyang Rd., Hualien 97002, Taiwan; 101327102@gms.tcu.edu.tw; 2 Department of Physiology, School of Medicine, Tzu Chi University, No. 701, Sec. 3, Zhongyang Rd., Hualien 97004, Taiwan; ktyang@mail.tcu.edu.tw; 3 Department of Surgery, Hualien Tzu Chi Hospital, Buddhist Tzu Chi Medical Foundation, No. 707, Sec. 3, Zhongyang Rd., Hualien 97002, Taiwan; 110752101@gms.tcu.edu.tw (P.-C.T.); 103327102@gms.tcu.edu.tw (Y.-P.L.); 4 School of Medicine, Tzu Chi University, No. 701, Sec. 3, Zhongyang Rd., Hualien 97004, Taiwan; 105311122@gms.tcu.edu.tw; 5 Department of Life Sciences, Tzu Chi University, No. 701, Sec. 3, Zhongyang Rd., Hualien 97004, Taiwan; 106711117@gms.tcu.edu.tw; 6 Department of Surgery, School of Medicine, Tzu Chi University, No. 701, Sec. 3, Zhongyang Rd., Hualien 97004, Taiwan

**Keywords:** ferroptosis, myocardial ischemia/reperfusion injury, gossypol acetic acid, glutathione peroxidase 4

## Abstract

Myocardial ischemia/reperfusion (I/R) injury has been associated with ferroptosis, which is characterized by an iron-dependent accumulation of lipid peroxide to lethal levels. Gossypol acetic acid (GAA), a natural product taken from the seeds of cotton plants, prevents oxidative stress. However, the effects of GAA on myocardial I/R-induced ferroptosis remain unclear. This study investigated the ability of GAA to attenuate I/R-induced ferroptosis in cardiomyocytes along with the underlying mechanisms in a well-established rat model of myocardial I/R and isolated neonatal rat cardiomyocytes. H9c2 cells and cardiomyocytes were treated with the ferroptosis inducers erastin, RSL3, and Fe-SP. GAA could protect H9c2 cells against ferroptotic cell death caused by these ferroptosis inducers by decreasing the production of malondialdehyde and reactive oxygen species, chelating iron content, and downregulating mRNA levels of *Ptgs2*. GAA could prevent oxygen-glucose deprivation/reperfusion-induced cell death and lipid peroxidation in the cardiomyocytes. Moreover, GAA significantly attenuated myocardial infarct size, reduced lipid peroxidation, decreased the mRNA levels of the ferroptosis markers *Ptgs2* and *Acsl4*, decreased the protein levels of ACSL4 and NRF2, and increased the protein levels of GPX4 in I/R-induced ex vivo rat hearts. Thus, GAA may play a cytoprotectant role in ferroptosis-induced cardiomyocyte death and myocardial I/R-induced ferroptotic cell death.

## 1. Introduction

Ferroptosis, a recently identified form of regulated cell death, is characterized by an iron-dependent accumulation of lipid peroxides to lethal levels, resulting in oxidative damage to lipid bilayers; it differs from apoptosis, necroptosis, and autophagy in various ways [1]. Ferroptosis is known to function in several pathophysiological contexts including ischemia/reperfusion injury (IRI)-associated diseases [2,3,4,5]. Recent studies have shown that ferroptosis inhibitors, such as liproxstatin-1 (Lip-1) or ferrostatin-1 (Fer-1), and iron chelators, such as deferoxamine and dexrazoxane, can attenuate myocardial infarct size during cardiac ischemia/reperfusion (I/R) via suppression of ferroptosis [6,7,8].

In addition, some studies have shown that Glutathione peroxidase 4 (GPX4) is also related to the protection of myocardium against IRI. GPX4 is a phospholipid hydroperoxidase that protects cells against membrane lipid peroxidation and plays a pivotal role as a regulator of ferroptosis [9]. Myocardial IRI increases the generation of oxidized phosphatidylcholines, which suppresses GPX4 activity and leads to ferroptotic cell death [10,11]. Feng et al. [6] showed that Lip-1 protects the mouse myocardium against IRI by increasing levels of GPX4 protein and reducing reactive oxygen species (ROS) levels to suppress ferroptosis. In another report, deferoxamine was shown to attenuate IRI-induced ferroptosis by reducing lipid peroxidation, iron content, and reversing GPX4 protein levels in rat hearts [8].

Experimental and clinical researchers continue to seek therapeutic approaches that can improve the IRI condition; however, the outcome of clinical treatment is often unsatisfactory, probably because ferroptosis caused by I/R is not reduced by such treatments. Gossypol acetic acid (GAA), a natural product obtained from the seeds of cotton plants, has been shown to prevent oxidative stress-induced necrosis in retinal pigment epithelial cells and anti-lipid peroxidation in the hepatic tissues of male rats [12,13,14]. In addition, GAA chelates iron and enhances total iron-binding capacity [15]. However, in studies of the heart, the benefits of GAA in I/R-induced ferroptosis remain unknown.

In the present study, H9c2 cardiomyoblast cells were first used and treated with ferroptosis inducers, including erastin (Era), (1S,3R)-RSL3 (RSL3), and chlorido [N, N-disalicylidene-1,2-phenylenediamine] iron (III) (Fe-SP). We confirm these inducers can effectively induce ferroptosis of H9c2 cells and clarify whether GAA have a protective effect against Era-, RSL3-, and Fe-SP-induced ferroptosis. Moreover, neonatal cardiomyocytes were used in oxygen-glucose deprivation/reperfusion (OGD/R) model, and the Langendorff isolated perfused rat heart model of global ischemia-reperfusion injury was used to investigate whether GAA can inhibit cardiac ferroptosis caused by OGD/R and I/R and to evaluate the possible mechanism of GAA’s protective effect.

## 2. Materials and Methods

### 2.1. Chemicals 

GAA was obtained from Selleck (Houston, TX, USA). Dimethyl sulfoxide (DMSO) and 1,10-Phenanthrolin (phen) were obtained from Sigma-Aldrich (St. Louis, MO, USA). Era, RSL3, Fe-SP, Fer-1, Lip-1, and Necrostatin-1 (Nec-1) were obtained from Cayman Chemical Company (Ann Arbor, MI, USA). ZVAD-FMK was obtained from BioVision (Milpitas, CA, USA).

### 2.2. H9c2 Cardiomyoblast Cell Culture

H9c2 cells were cultured in Dulbecco’s modified Eagle medium (DMEM)-high glucose (Gibco, Grand Island, NY, USA) with 1% penicillin-streptomycin (Gibco) and 10% fetal bovine serum (Gibco). When H9c2 cells reached 80% confluence, they were rinsed with phosphate-buffered saline (PBS) solution, detached using 0.25% trypsin-EDTA (Gibco), and then neutralized by adding fresh medium. The cell suspension was centrifuged at 1200 rpm for 5 min. H9c2 cells were then seeded into 15-cm Petri dishes at a density of 2 × 10^4^ cells/cm^2^ and maintained at 37 °C in a humidified atmosphere containing 5% CO_2_. The medium was changed every 2 days.

### 2.3. Preparation and Culture of Ventricular Myocytes

Two-day-old Sprague–Dawley rats (both sexes) were sacrificed by decapitation and their ventricles were minced into small pieces. The cardiac tissues were digested using 0.045% pancreatin (Sigma-Aldrich) and 0.01% collagenase II (Gibco) in Hank’s solution (Gibco) before being incubated with F-12 medium (80% F-12 nutrient mixture, 10% horse serum, 10% fetal bovine serum, and 1% penicillin/streptomycin; Gibco) to inactivate enzymatic digestion. Cells were plated onto a 10-cm dish for 1 h at 37 °C in a 5% CO_2_ incubator to remove fibroblasts. The cardiomyocytes were seeded on a 0.1% collagen-coated 6-cm dish in F-12 medium before 10-μM cytosine arabinoside was added to inhibit fibroblast proliferation. The medium was replaced every day during the experiments. The procedures and protocols were approved by the Institution of Animal Care and Use Committee of the Tzu Chi University (IACUC Approval No. 110028). This culture protocol generally requires pooling of 7–10 neonatal hearts for a single replicate. All experiments were independently repeated 4 times or more for cell culture, and a total of 150 neonatal rats were used.

To mimic the I/R injury in the rat heart in vivo, OGD/R was performed in the cardiomyocytes; the cells were incubated in serum- and glucose-free DMEM (Sigma-Aldrich) in an anoxic environment (95% N_2_ and 5% CO_2_) for 6 h at 37 °C. Immediately after the OGD, the cells were reperfused by removing the medium and replacing DMEM containing 10-mM glucose, 10% horse serum, 10% fetal bovine serum, and 1% penicillin/streptomycin and were then incubated for 24 h at 37 °C in a 5% CO_2_ incubator. The control was incubated with DMEM for 30 h at 37 °C in a 5% CO_2_ incubator.

### 2.4. Langendorff Heart Perfusion System

A total of 55 adult male Sprague-Dawley rat (350–450 g) were anesthetized with urethane (1.5 g/kg, i.p.), then the hearts were perfused in a Langendorff system. After 30 min of stabilization, hearts were subjected to 30 min of global no-flow ischemia by stopping the perfusion. Reperfusion was followed with Krebs Henseleit (KH) buffer and GAA together for 2 h. A thermoregulated chamber kept the heart at 37 °C throughout the experiment. Control hearts were not subjected to I/R. The heart slices were sectioned at a thickness of 2 mm and stained with triphenyltetrazolium chloride (25 mg/100 mL) for 10 min and then fixed with 4% formaldehyde solution for 48 h to enhance color contrast. Unstained areas were measured using ImageJ software (Version 1.50; National Institutes of Health, Bethesda, MD, USA) to calculate the percentage of grayish-white areas. 

### 2.5. Cell Viability Assay

H9c2 cells and cardiomyocytes were seeded onto 24-well plates for 16-h and 24-h treatments, respectively. After, the medium was removed from the cells. Subsequently, 3-(4,5-dimethylthiazol-2-yl)-2,5-diphenyl tetrazolium bromide (MTT) (Sigma-Aldrich) solution (5 mg/mL in PBS) was added to each 24-well plate for 2 h. After MTT was removed, the precipitate was solubilized in 500 μL of DMSO. The absorbance of each well at 570 nm was measured using an enzyme-linked immunosorbent assay (ELISA) reader (Multiskan EX; Thermo, Waltham, MA, USA). 

### 2.6. Iron Content Analysis

H9c2 cells were collected and washed twice with PBS before being stained with 2-μM of Phen Green SK (PGSK) diacetate (excitation/emission = 507/532) (Cayman Chemical Company); the fluorescence of the PGSK dye is quenched upon interaction with Fe^2+^. The cell pellets were resuspended in 1 mL of N-2-hydroxy-ethylpiperazine-N′-2-ethanesulphonic acid (HEPES)-buffered Tyrode solution (NT), the cell suspensions were transferred to disposable fluorescence-activated cell sorting tubes, and the fluorescence profiles of the samples were assessed using a Gallios^TM^ Flow Cytometer (Beckman Coulter, Brea, CA, USA). Data were collected from the FL1 detector and mean fluorescence intensities were compared between treated groups. A minimum of 10,000 cells were analyzed per condition.

### 2.7. Cell Death Analysis

An Annexin V-FITC Apoptosis Detection Kit (excitation/emission = 488/530) (Bio-Vision) was used to visualize the dead cells within cardiomyocytes after ferroptosis inducer or GAA treatments. Fluorescence was detected with the Gallios^TM^ Flow Cytometer; data were collected from a FL1 detector, whereas propidium iodide (PI) (excitation/emission = 536/617) was collected from a FL3 detector. A minimum of 10,000 H9c2 cells and 5000 cardiomyocytes were analyzed per condition. 

### 2.8. ROS Analysis

H9c2 cells (1 × 10^4^ cells/cm^2^) were treated with Era (1 μM), RSL3 (0.1 μM) or Fe-SP (0.5 μM) with or without GAA (2 μM) for 10 h. After these 10-h treatments, H9c2 cells were washed with NT and suspended in NT containing 2.5-μM CM-H_2_DCFDA (excitation/emission = 495/529) (Molecular Probes, Eugene, OR) for 30 min at 37 °C. Subsequently, ROS production was detected using the Gallios^TM^ Flow Cytometer. Data were collected from the FL1 detector and mean fluorescence intensities were compared between treated groups. A minimum of 10,000 cells were analyzed per condition. 

### 2.9. Lipid Peroxidation Analysis

#### 2.9.1. Thiobarbituric Acid Reactive Substances (TBARS) Assay

Lipid peroxidation was measured using a TBARS Assay Kit (Cayman Chemical Company) via malondialdehyde (MDA) formation. H9c2 cells (3.5 × 10^4^ cells/cm^2^) were treated with Era (1 μM), RSL3 (0.1 μM), or Fe-SP (0.5 μM) with or without GAA (2 μM) for 16 h. After these 16-h treatments, cells were washed with PBS before being collected and sonicated. Thiobarbituric acid sodium dodecyl sulfate solution was then added with a color reagent forcefully down the side of each vial. Vials were added to vigorously boiling water for one hour. After, vials were incubated on ice for 10 min, then centrifuged at 1600× *g* for 10 min. Fluorescence intensity was measured using an ELISA plate reader (Multiskan EX; Thermo) with 530- and 555-nm excitation and emission filters, respectively. 

#### 2.9.2. Confocal Images

On the day before the experiment, cardiomyocytes (6 × 10^4^ cells/cm^2^) were seeded in 24-mm 0.1% collagen-coated coverslips. On the day of the experiment, cells were treated with Fe-SP for 2 h and then incubated with NT containing 2-μM C11 BODIPY 581/591 (Cayman Chemical Company) for 1 h at room temperature. C11 BODIPY 581/591 is a fluorescent indicator of lipid oxidation. Oxidation of the polyunsaturated butadienyl portion of the dye results in a shift in the fluorescence of the excitation peak from 581 to 500 nm and the emission peak from 591 nm (red) to 510 nm (green). Images were captured at 40× magnification using a confocal microscope (Nikon C2 Si^+^) and analyzed using NIS-Elements imaging software. The following excitation and emission settings were used: excitation: 488 nm, emission: 510–574 nm; excitation: 568 nm, emission: 574–613 nm.

### 2.10. Western Blot Analysis

Cells or heart tissue were homogenized using a RIPA lysis buffer (Millipore, Burlington, MA, USA) containing 1% protease inhibitor (Calbiochem) and 0.5% phosphatase inhibitor (Calbiochem). The lysates were centrifuged at 12,000 rpm at 4 °C for 15 min and then protein concentrations were determined using a BSA Protein Assay Kit (Bio-Red, Hercules, CA, USA). A western blot analysis was performed according to a previous report [16]. Briefly, the samples containing 30 μg of proteins were electrophoresed on 10% SDS-PAGE gels and transferred to polyvinylidene difluoride membranes. These membranes were blocked using 5% non-fat milk before being incubated with the following primary antibodies overnight at 4 °C: anti-NRF2 (GTX103322; GeneTex; 1:1000), anti-GPX4 (GTX54095; GeneTex; 1:1000), anti-ACSL4 (Ab155282; Abcam; 1:5000), anti-4-HNE (ARG23967; arigo; 1:1000), and anti-GAPDH (GTX100118; GeneTex; 1:10,000). Subsequently, the membranes were incubated with secondary antibodies for 2 h. The blots were visualized using an ECL western blotting reagent (GE Healthcare, Uppsala, Sweden), analyzed using Image-Pro Plus software, and normalized using GAPDH. 

### 2.11. Quantitative Real-Time Polymerase Chain Reaction

Total RNA was isolated with TRIzol reagent (Ambion, Carlsbad, CA); 3 µg of total RNA was used to synthesize cDNA with a Verso^TM^ cDNA Kit (Thermo). Real-time polymerase chain reaction (PCR) was performed using the LightCycler 480 system (Roche, Basel, Switzerland) with Fast SYBR Green Master Mix (Thermo). The primer sequences used for PCR were as follows: prostaglandin-endoperoxide synthase 2 (*Ptgs2*) forward: 5′- ATG TTC GCA TTC TTT GCC CAG -3′; *Ptgs2* reverse: 5′- TAC ACC TCT CCA CCG ATG AC -3′; *Acls4* forward: 5′-TCC AAG CCA GAA AAC TCA AGC-3′; *Acls4* reverse: 5′-GGT GTA CAT GAC AAT GGC CAT-3′; *Gapdh* forward: 5′-ATG TTC CAG TAT GAC TCC ACT CAC G-3′; *Gapdh* reverse: 5′-GAA GAC ACC AGT AGA CTC CAC GAC A-3′. The amplification conditions for the PCR were as follows (in a total volume of 25 μL): 55 cycles of denaturation at 65 °C for 5 min, primer annealing at 50 °C for 60 min, and extension at 70 °C for 15 min. *Ptgs2* and *Acsl4* mRNA expression levels were normalized using *Gapdh* as a housekeeping gene. All gene expression was analyzed using the comparative Ct method (2^−ΔΔCt^): ΔΔCt = ΔCt (experimental sample) − ΔCt (positive control), where ΔCt = Ct of target gene—Ct of internal reference gene. Ct indicates the number of amplification cycles that the reaction’s real-time fluorescence intensity needs to pass when it reaches a set detection threshold. *Gapdh* mRNA levels were used as internal controls to normalize the results. 

### 2.12. Statistics

All experiments were conducted at least three times, and the data are expressed as means ± standard error of the mean (SEM). Comparisons were subjected to one-way analysis of variance followed by Fisher’s least significant difference test. *p* values < 0.05 were considered significant.

## 3. Results

### 3.1. Effects of GAA on Ferroptotic Cell Death in H9c2 Cardiomyoblast Cells

**Figure 1:**
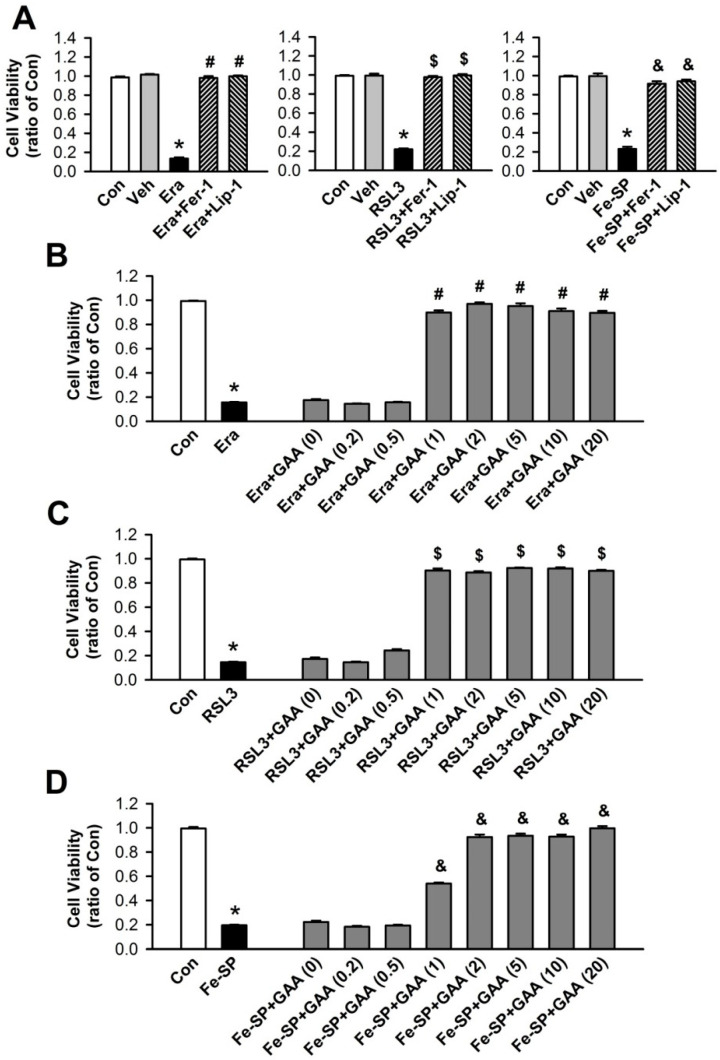
GAA prevents ferroptotic cell death in H9c2 cardiomyoblast cells. (**A**) MTT results showing a significant decrease in cell viability at 16-h postexposure to 1-μM Era (left panel), 0.1-μM RSL3 (central panel), and 0.5-μM Fe-SP (right panel); these effects were rescued by 1-μM Fer-1 and 2-μM Lip-1 (n = 12). GAA (1–20 μM) treatment abolished (**B**) Era-, (**C**) RSK3-, and (**D**) Fe-SP-induced cell viability inhibition (n = 12). In each group, 3 samples were analyzed, and the same experiment was assessed by repeating cell culture four times. All data represent means ± SEM. * *p* < 0.05 versus the control group; # *p* < 0.05 versus the Era; $ *p* < 0.05 versus RSL3; & *p* < 0.05 versus Fe-SP. Con, control; Veh, vehicle; Era, erastin; Fer-1, ferrostatin-1; Lip-1, liproxstatin-1; RSL3, (1S,3R)-RSL3; Fe-SP, chlorido [N, N-disalicylidene-1,2-phenylenediamine] iron (III); GAA, gossypol acetic acid.

H9c2 cells are widely accepted as a valid in vitro system to study cardiac disease. To investigate the ferroptotic cell death in H9c2 cells, we used MTT assay to measure cell viability. First, we used a ferroptosis inducer, including Era (1 μM), RSL3 (0.1 μM), and Fe-SP (0.5 μM) to induce cell death. The results showed that Era, RSL3, and Fe-SP significantly reduced cell viability. To confirm that the decrease in cell viability is related to ferroptosis, we used ferroptosis inhibitors, Fer-1 (1 μM) and Lip-1 (2 μM). The ferroptosis inhibitors significantly reduced Era-, RSL3-, and Fe-SP-induced cell death (Figure 1A). Therefore, Era, RSL3, and Fe-SP can be used to induce ferroptotic cell death in H9c2 cells. DMSO (1:1000), the vehicle used to dissolve Era, RSL3, and Fe-SP, did not significantly affect the viability of H9c2 cells. We also used Era-, RSL3-, and Fe-SP-induced ferroptotic cell death with and without various GAA concentrations (0.2–20.0 μM) in a MTT assay to determine whether GAA could inhibit this process. Similar to the ferroptosis inhibitors, GAA (1–20 μM) inhibited ferroptotic cell death in H9c2 cells (Figure 1B–D).

**Figure 2:**
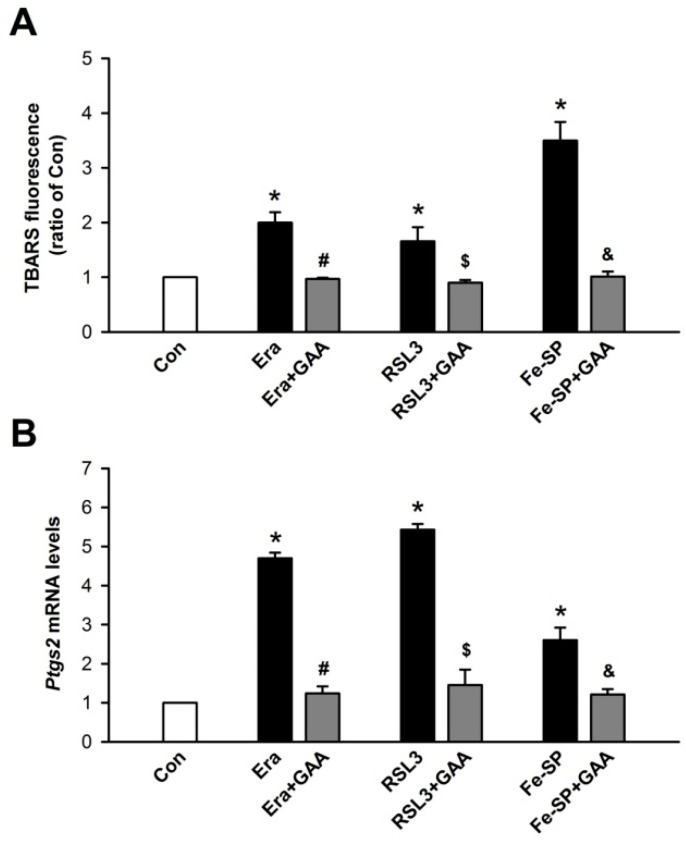
GAA inhibits lipid peroxidation and ferroptosis markers in H9c2 cardiomyoblast cells. (**A**) Results of a TBARS assay showing that GAA (2 μM) inhibits 1-μM Era-, 0.1-μM RSL3-, and 0.5-μM Fe-SP-induced lipid peroxidation (n  =  4). (**B**) GAA significantly decreased the levels of *Ptgs2* mRNA after exposure to Era, RSL3, and Fe-SP (n  =  4). The same experimental group was assessed by repeating cell culture 4 times. All data represent means ± SEM. * *p* < 0.05 versus the control group; # *p* < 0.05 versus Era; $ *p* < 0.05 versus RSL3; & *p* < 0.05 versus Fe-SP. Con, control; Era, erastin; Fer-1, ferrostatin-1; Lip-1, liproxstatin-1; RSL3, (1S,3R)-RSL3; Fe-SP, chlorido [N, N-disalicylidene-1,2-phenylenediamine] iron (III); GAA, gossypol acetic acid.

**Figure 3:**
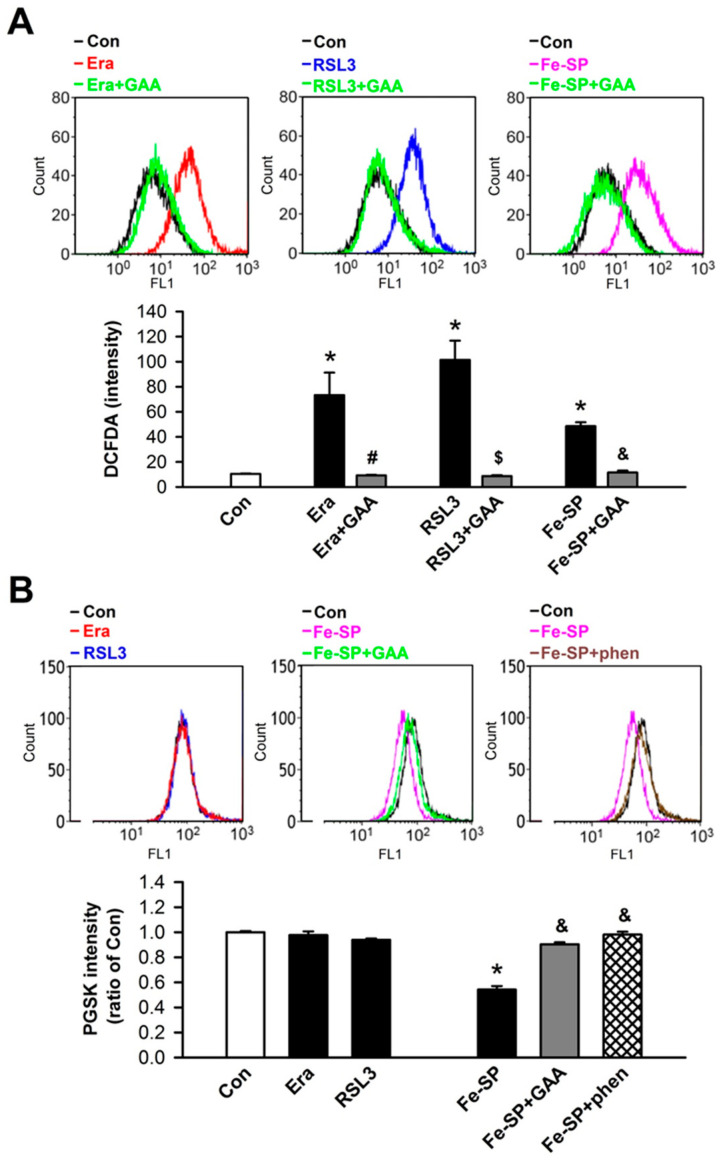
GAA reduces ROS and iron accumulation in H9c2 cardiomyoblast cells. (**A**) Treatment with 1-μM Era, 0.1-μM RSL3, and 0.5-μM Fe-SP for 10 h significantly enhanced intracellular ROS in H9c2 cells; these effects were decreased by GAA (2 μM). Top panel: intracellular ROS levels were assessed by flow cytometry analysis using a CM-H_2_DCFDA probe (an indicator of intracellular ROS); bottom panel: quantitative data (n  =  4). (**B**) Fe-SP, but not Era or RSL3, significantly increased intracellular iron content; this effect was abolished by 2-μM GAA or 50-μM 1, 10-phenanthroline (phen; a ferrous iron chelator). The bottom panel shows data obtained from analysis of the top panel (n  =  4). For each flow cytometric analysis, 10,000 cells were counted and plotted. Data are shown as overlapping histogram plots. The same experimental group was assessed by repeating cell culture 4 times. All data represent means ± SEM. * *p* < 0.05 versus the control group; # *p* < 0.05 versus Era; $ *p* < 0.05 versus RSL3; & *p* < 0.05 versus Fe-SP. Con, control; Era, erastin; Fer-1, ferrostatin-1; Lip-1, liproxstatin-1; RSL3, (1S,3R)-RSL3; Fe-SP, chlorido [N, N-disalicylidene-1,2-phenylenediamine] iron (III); GAA, gossypol acetic acid; phen, 1, 10-phenanthroline.

Ferroptosis is a form of regulated cell death that is dependent on intracellular iron and ROS accumulation; it is characterized by lipid peroxidation. To test whether GAA (2 μM) could inhibit Era-, RSL3-, and Fe-SP-induced lipid peroxidation in H9c2 cells, a TBARS assay was used to detect the level of MDA in lysates. At 16 h after ferroptosis inducers and GAA cotreatments in H9c2 cells, GAA was found to attenuate Era-, RSL3-, and Fe-SP-induced an increase in lipid peroxidation in these cells (Figure 2A). To verify GAA inhibition of ferroptosis, we detected the mRNA levels of *Ptgs2*, a molecular marker of ferroptosis, via real-time PCR. Notably, GAA reduced Era-, RSL3-, and Fe-SP-induced upregulation of the *Ptgs2* mRNA levels in H9c2 cells (Figure 2B). To determine whether GAA (2 μM) could also inhibit ferroptosis inducer-mediated ROS and iron enhancements, we used flow cytometry analysis along with CM-H_2_DCFDA, an indicator of ROS (Figure 3A, upper panel), and an iron indicator dye, PGSK (of which the fluorescence is quenched by intracellular labile iron pools; Figure 3B, upper panel), respectively. After cotreatment with GAA for 10 h substantially reduced Era-, RSL3-, or Fe-SP-induced ROS accumulation in H9c2 cells (Figure 3A, bottom panel). Neither Era nor RSL3 increased iron content in H9c2 cells. In contrast, addition of Fe-SP to these cells resulted in a loss of PGSK fluorescence intensity, suggesting Fe-SP increased intracellular iron concentration (Figure 3B, bottom panel). In addition, to confirm that the decrease in PGSK fluorescence intensity is caused by the increase in iron concentration; thus, 50-μM phen (a ferrous ion chelator) was applied to inhibit intracellular iron accumulation in the Fe-SP-treated H9c2 cells. In summary, 2-μM GAA protects H9c2 cardiomyoblast cells from ferroptosis inducer-mediated iron accumulation, ROS accumulation, and lipid peroxidation.

### 3.2. Effects of GAA on Ferroptosis-Induced Neonatal Rat Myocardial Cell Death

**Figure 4:**
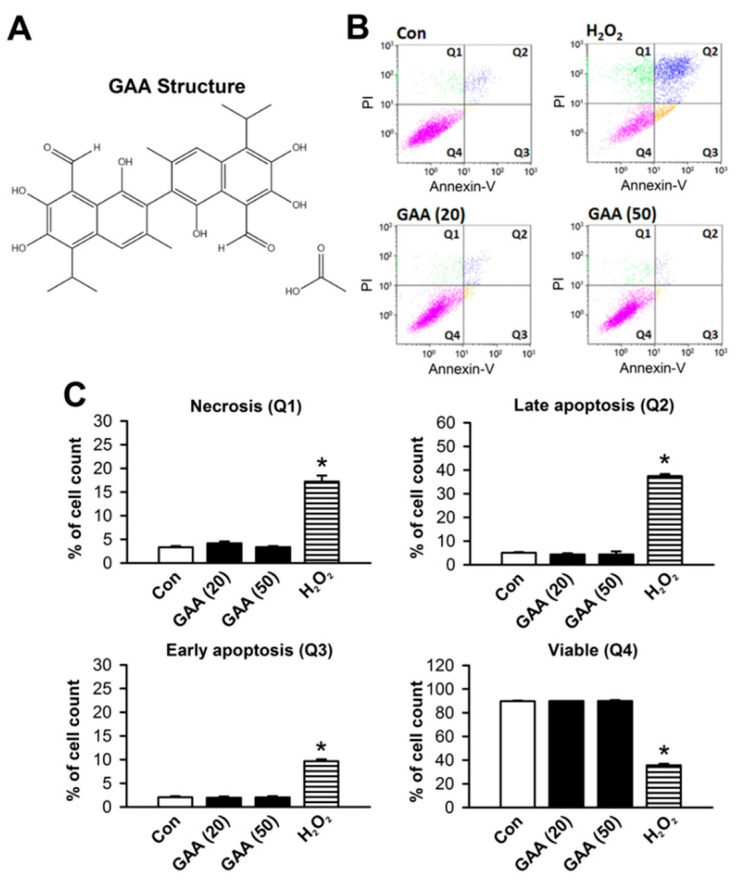
GAA does not induce cell death in neonatal rat cardiomyocytes. (**A**) The chemical structure of GAA. (**B**) GAA did not induce apoptosis and necrosis according to analysis by flow cytometry of Annexin V-FITC/PI double staining at 24 h. H_2_O_2_ was used as a positive control. (**C**) Statistical analysis of necrosis (Q1), late apoptosis (Q2), early apoptosis (Q3), and viability (Q4) in neonatal rat cardiomyocytes (n = 4). The same experimental group was assessed by repeating cell culture four times. All data represent means ± SEM. * *p* < 0.05 versus the control group. Con, control; GAA, gossypol acetic acid; H_2_O_2_, hydrogen peroxide.

**Figure 5:**
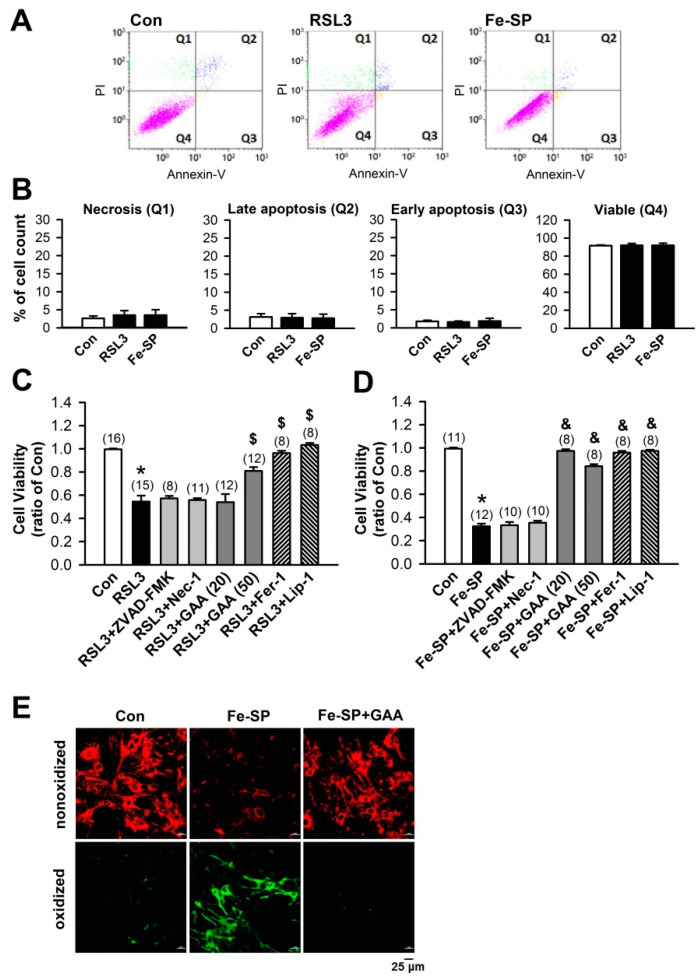
GAA protects against ferroptotic cell death and inhibits lipid peroxidation in neonatal rat cardiomyocytes. (**A**) RSL3 and Fe-SP did not induce apoptosis and necrosis according to analysis by flow cytometry of Annexin V-FITC/PI double staining at 24 h. (**B**) Statistical analysis of necrosis (Q1), late apoptosis (Q2), early apoptosis (Q3), and viability (Q4) in neonatal rat cardiomyocytes (n = 4). (**C**) MTT results showing a significant decrease in cell viability 24 h postexposure to 0.5-μM RSL3, abolished by GAA (20–50 μM), Fer-1 (1 μM), and Lip-1 (2 μM), but not ZVAD-FMK (10 μM) or Nec-1 (10 μM). Values shown in parenthesis represent the number in each group. In each group, 2 to 3 samples were analyzed, and the same experiment was assessed by repeating cell culture four times or more. (**D**) MTT results showing a significant decrease in cell viability 24 h postexposure to 2-μM Fe-SP, abolished by GAA (50 μM), Fer-1 (1 μM), and Lip-1 (2 μM), but not ZVAD-FMK (10 μM) or Nec-1 (10 μM). Values shown in parenthesis represent the number in each group. In each group, 2 to 3 samples were analyzed, and the same experiment was assessed by repeating cell culture four times or more. (**E**) Confocal images of neonatal rat cardiomyocytes treated with Fe-SP (2 μM) for 2 h; a significant increase in levels of lipid peroxidation was observed (green), but this effect was abolished by 50-μM GAA. Red, nonoxidized form of C11 BODIPY; Green, oxidized form of C11 BODIPY. Scale bars: 25 μm. All data represent means ± SEM. * *p* < 0.05 versus the control group; $ *p* < 0.05 versus RSL3; & *p* < 0.05 versus Fe-SP. Con, control; Fer-1, ferrostatin-1; Lip-1, liproxstatin-1; RSL3, (1S,3R)-RSL3; Fe-SP, chlorido [N, N-disalicylidene-1,2-phenylenediamine] iron (III); GAA, gossypol acetic acid; Nec-1, necrostatin-1.

Annexin V-FITC and PI assay was used to check the cytotoxicity of GAA in neonatal cardiomyocytes. GAA is a crystallized form of gossypol (Figure 4A); it is the most biologically active form of this phenol [17]. Previous studies have shown that gossypol induces cell death in cancer cells [18,19] and germline stem cells [20]; hence we used primary neonatal rat cardiomyocytes cultured in vitro and treated with GAA (20 and 50 μM) to assess the induction of cell death via flow cytometry analysis with Annexin V-FITC and PI for apoptosis and necrosis detection, respectively (Figure 4B). GAA did not cause significant necrosis (Q1), late apoptosis (Q2), or early apoptosis (Q3) relative to the positive control, i.e., 20-μM H_2_O_2_ (Figure 4C). RSL3 (0.5 μM) and Fe-SP (2 μM) treatments were also used to verify the occurrence of ferroptosis and exclude the occurrence of necrosis or apoptosis. As shown in Figure 5A,B, RSL3 and Fe-SP did not significantly affect the number of necrotic cells, early and late apoptotic cells, or viable cells. In the Supplementary Materials, we found that higher doses of RSL3 (2 μM) and Fe-SP (10 μM) can cause apoptosis and necrosis in cardiomyocytes. In addition, we used several cell death inhibitors to address the possible involvement of other cell death pathways and an MTT assay to examine cell viability. As shown in Figure 5C,D, when we treated cardiomyocytes with RSL3 (0.5 μM) or Fe-SP (2 µM) for 24 h in the presence or absence of Fer-1 (1 µM) or Lip-1 (2 µM), cell death was prevented. However, RSL3- or Fe-SP-induced cell death was not inhibited by ZVAD-FMK (10 μM) or Nec-1 (10 μM). Therefore, RSL3- and Fe-SP-induced cardiomyocyte death was because of ferroptosis but did not involve apoptosis or necroptosis. Similar to the ferroptosis inhibitors, GAA significantly improved ferroptotic cell death in RSL3- and Fe-SP-treated cardiomyocytes. To confirm the effect of GAA on ferroptosis, we used the lipid ROS probe C11 BODIPY 581/591 and performed confocal microscopy observations. Cotreatment with Fe-SP (2 µM) and GAA (50 µM) significantly reduced lipid peroxidation in the cardiomyocytes (Figure 5E). These results indicate that GAA (20 and 50 µM) could abolish Fe-SP-induced ferroptotic cell death and lipid peroxidation but did not induce cytotoxicity in the cardiomyocytes.

**Figure 6:**
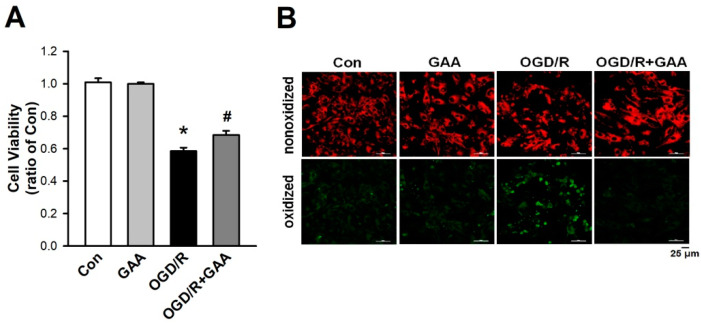
GAA reduces OGD/R -induced cell death and inhibits lipid peroxidation in neonatal rat cardiomyocytes. (**A**) Neonatal rat cardiomyocytes were exposed to OGD/R, and cell survival was determined 24 h after OGD/R using the MTT assay. The results show that OGD/R leads to significant cell viability reduction. This effect was rescued by a posttreatment with 5-μM GAA (n = 4). The same experimental group was assessed by repeating the cell culture four times. (**B**) Confocal images of neonatal rat cardiomyocytes treated with OGD/R. A significant increase was observed in the level of lipid peroxidation (green), but this effect was abolished by a post-treatment with 5-μM GAA. Red, nonoxidized form of C11 BODIPY; Green, oxidized form of C11 BODIPY. Scale bars: 25 μm. All data are represented as the means ± SEM. * *p* < 0.05 versus the control group; # *p* < 0.05 versus OGD/R. Con, control; GAA, gossypol acetic acid; OGD/R, oxygen-glucose deprivation/reperfusion.

In addition, we used the OGD/R to mimic the myocardial I/R in vitro. First, we established that GAA (5 µM) posttreatment significantly reduced OGD/R-induced cell death in the cardiomyocytes (Figure 6A). Second, we examined the effect of GAA on lipid peroxidation using the lipid ROS probe C11 BODIPY 581/591 and confocal microscopy. As shown in Figure 6B, GAA post-treatment prevented the OGD/R-induced lipid peroxidation in the cardiomyocytes. Taken together, GAA not only protects cardiomyocytes against ferroptosis inducers-induced ferroptotic cell death, but also protects against ferroptotic cell death induced by OGD/R.

### 3.3. GAA Attenuates I/R-Induced Ferroptosis in Ex Vivo Rat Hearts

**Figure 7:**
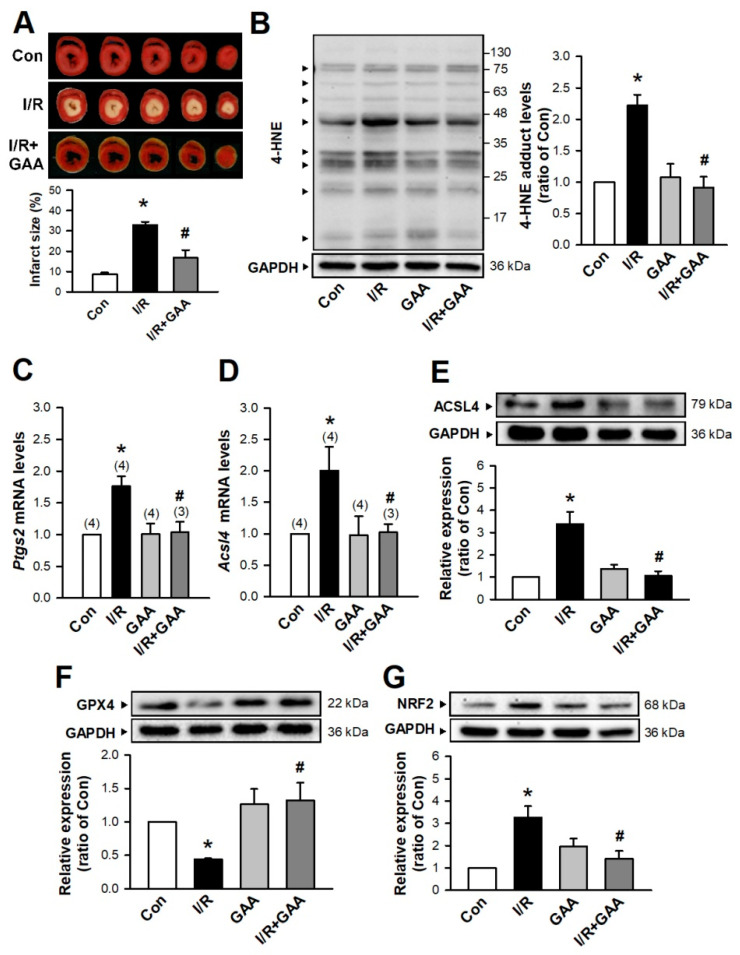
The mechanism by which GAA attenuates myocardial I/R-induced ferroptotic cell death in ex vivo rat hearts. (**A**) Representative images (Top) and quantitative data (Bottom) showed that GAA (5 μM) significantly reduced the I/R-induced increase in myocardial infarct size (n = 3). (**B**) Cotreatment with I/R and GAA (10 μM) significantly decreased the 4-HNE adduct levels (n = 4). GAA significantly decreased levels of *Ptgs2* (**C**) and *Acsl4* (**D**) mRNA in myocardial ischemia-treated hearts according to quantitative PCR analysis. Values shown in parenthesis represent the number in each group. (**E**) Treatment with GAA significantly inhibited the I/R-increased levels of ACSL4 protein enhancement (n = 9). (**F**) Treatment with GAA increased GPX4 protein levels compared with those observed in the I/R-treated group (n = 9). (**G**) Treatment with GAA significantly inhibited I/R-increased levels of NRF2 protein (n = 6). All data represent means ± SEM. * *p* < 0.05 versus the control group; # *p* < 0.05 versus I/R. Con, control; I/R, ischemia/reperfusion; GAA, gossypol acetic acid.

Whether GAA could protect against IRI remained unknown. We examined isolated Langendorff-perfused hearts that were subjected to 30 min of no-flow ischemia followed by 2 h reperfusion. TTC staining was used to assess myocardial tissue viability and determine myocardial infarct size. Our results showed that post-treatment with GAA (5 μM) significantly attenuated I/R-induced cardiac infarct size in ex vivo rat hearts (Figure 7A). Cardiac IRI induces various types of cell death including necrosis, apoptosis, and ferroptosis. Therefore, we investigated whether GAA protects against I/R-induced cardiac ferroptosis. As shown in Figure 7B, the GAA (10 µM) treatment significantly reduced the lipid peroxidation products, 4-hydroxynonenal (4-HNE) in cardiac I/R. As shown in Figure 7C,D, cotreatment with I/R and GAA significantly reduced *Ptgs2* and *Acsl4* mRNA levels relative to the levels detected upon the I/R treatment alone. In addition, GAA treatment decreased ACSL4 protein levels in cardiac I/R (Figure 7E). We explored the mechanism by which GAA inhibited I/R-induced ferroptosis in cardiomyocytes by measuring GPX4 protein levels via western blotting. The I/R treatment significantly decreased GPX4 protein levels, but this effect was prevented by cotreatment with GAA (Figure 7F). Previous studies have associated I/R with activation of nuclear factor-erythroid 2-related factor 2 (NRF2) transcription factor [21,22,23]. Thus, activation or inhibition of NRF2 as potential causes of the ferroptosis-inhibition effects of GAA was evaluated. Cotreatment with I/R and GAA significantly decreased NRF2 protein levels relative to the levels observed with I/R treatment alone (Figure 7G). These results indicate that GAA decreases lipid peroxidation, maintains the protein levels of GPX4 and NRF2, and thereby attenuates I/R-induced ferroptotic cell death in ex vivo rat hearts.

## 4. Discussion

Gossypol is reported to possess antifertility, antitumor, and antioxidant properties [24]. However, GAA has been used as a drug form of gossypol because the latter is prone to oxidization when exposed to air. Currently, abundant evidence has indicated that GAA is a promising novel anticancer drug [25,26]. In the present study, we showed that GAA (1–20 µM) can protect H9c2 cells from ferroptosis inducer-mediated cell death. Thus, GAA has protective activity in H9c2 cells at concentrations 2.5–50.0-fold lower than those used against cancer cells. A previous study showed that gossypol at <5-µM did not induce cell death in germline stem cells [20]. In addition, Hanus et al. [12] reported that 5-µM GAA could significantly inhibit oxidative stress-induced retinal pigment epithelial death. In the present study, GAA at 20- or 50-µM could abolish Fe-SP-induced ferroptotic cell death but did not affect cell death in cardiomyocytes. In another study, gossypol was shown to significantly decrease total plasma cholesterol and low-density lipoprotein levels but not high-density lipoprotein levels in adult male cynomolgus monkeys [24]. In our study, 5- or 10-µM GAA treatment reduced I/R-induced *Ptgs2* and *Acsl4* mRNA level increases while also attenuating I/R-induced ferroptotic cell death in ex vivo rat hearts. Given that the definitive concentration at which GAA presents a risk to human health is yet to be identified, this matter requires further study.

Many previous results have shown that after OGD/R treatment of primary cultured cardiomyocytes or H9c2 cells, intracellular Fe^2+^, ROS, and MDA have a significant increase. Moreover, the increase in ACSL4 and the decrease in GPX4 have been observed, indicating that OGD/R can trigger cardiac cells ferroptosis [27,28,29,30]. ROS can cause many different types of cell damage and death. Therefore, the study found that OGD/R induces cell ferroptosis, and different cell deaths can also be observed, such as apoptosis [28,29,30] or autophagy [27]. In our previous study, although GAA significantly inhibits OGD/R-induced cell lipid peroxidation and cell death, the improvement of cell viability cannot achieve the effect of inhibiting lipid peroxidation. It should be the GAA’s inability to completely inhibit other OGD/R-induced forms of cell death and the signaling pathway of injury.

Previous studies have shown that GAA could potentially inhibit oxidative stress and lipid peroxidation [12,14]. We found that GAA inhibited ferroptosis inducer-mediated MDA and ROS enhancements in H9c2 cells, as effectively as Fe-SP- and OGD/R-induced lipid peroxidation in the cardiomyocytes. In addition, we investigated the mechanism through which GAA prevents I/R-induced ferroptotic cell death in ex vivo rat hearts. GAA increased the protein levels of GPX4, a glutathione peroxidase that reduces lipid peroxides and improves cell survival in I/R-induced ex vivo rat hearts.

NRF2 is an emerging regulator of cellular resistance to oxidative stress. Under normal conditions, NRF2 is quickly degraded, mainly via the Kelch-like ECH-associated protein 1-mediated pathway. Under oxidative stress, NRF2 is not degraded but instead translocated from the cytoplasm to the nucleus where it binds to the antioxidant response elements and regulates the transcription of antioxidant genes that encode products such as heme oxygenase 1 (HO-1) [31], which mediates protection against IRI [32]. However, some studies have indicated that HO-1 plays a dominant role in ferroptosis, possibly in association with iron accumulation [7,33]. GAA is a powerful chelator of iron [15]. In the present study, GAA decreased the content of intracellular Fe^2+^ in Fe-SP-treated H9c2 cells. Moreover, myocardial I/R and GAA treatment combined were found to decrease NRF2 protein levels in ex vivo rat hearts. The underlying mechanism by which GAA controls the NRF2-HO-1 pathway to decrease intracellular Fe^2+^, subsequently attenuates lipid peroxides, and eventually protects against myocardial I/R-induced ferroptotic cell death was not fully revealed by the present work; thus, further study is required in this area.

## 5. Conclusions

In this study, we showed that GAA significantly reduces ROS, lipid peroxidation, and chelated iron in ferroptosis inducer-mediated cardiomyocyte death. Furthermore, we revealed that the GAA treatment of rat hearts effectively prevents myocardial I/R-induced lipid peroxidation and ferroptotic cell death through increased GPX4 protein levels. Therefore, GAA might have significant implications in the treatment of IRI-related heart diseases.

## Data Availability

All data and materials used/generated in this study are available from the corresponding author upon reasonable request.

## Supplementary Materials

The following are available online at https://www.mdpi.com/article/10.3390/biom11111667/s1, Figure S1: Treatment with RSL3 and Fe-SP in neonatal rat cardiomyocyte for 24 h and examined though confocal microscopy using Hoechst 33342 and PI stainings. The results showed that RSL3 (2 μM) and Fe-SP (10 μM) treatments increased the number of PI positive cells but not at low concentrations of RSL3 (0.5 μM) and Fe-SP (2 μM) in neonatal rat cardiomyocytes.

## References

[1] Li N., Jiang W., Wang W., Xiong R., Wu X., Geng Q. (2021). Ferroptosis and its emerging roles in cardiovascular diseases. Pharmacol. Res..

[2] Guan X., Li X., Yang X., Yan J., Shi P., Ba L., Cao Y., Wang P. (2019). The neuroprotective effects of carvacrol on ischemia/reperfusion-induced hippocampal neuronal impairment by ferroptosis mitigation. Life Sci..

[3] Li Y., Feng D., Wang Z., Zhao Y., Sun R., Tian D., Liu D., Zhang F., Ning S., Yao J. (2019). Ischemia-induced ACSL4 activation contributes to ferroptosis-mediated tissue injury in intestinal ischemia/reperfusion. Cell Death Differ..

[4] Su L., Jiang X., Yang C., Zhang J., Chen B., Li Y., Yao S., Xie Q., Gomez H., Murugan R. (2019). Pannexin 1 mediates ferroptosis that contributes to renal ischemia/reperfusion injury. J. Biol. Chem..

[5] Li W., Li W., Leng Y., Xiong Y., Xia Z. (2020). Ferroptosis is involved in diabetes myocardial ischemia/reperfusion injury through endoplasmic reticulum stress. DNA Cell Biol..

[6] Feng Y., Madungwe N.B., Imam Aliagan A.D., Tombo N., Bopassa J.C. (2019). Liproxstatin-1 protects the mouse myocardium against ischemia/reperfusion injury by decreasing VDAC1 levels and restoring GPX4 levels. Biochem. Biophys. Res. Commun..

[7] Fang X., Wang H., Han D., Xie E., Yang X., Wei J., Gu S., Gao F., Zhu N., Yin X. (2019). Ferroptosis as a target for protection against cardiomyopathy. Proc. Natl. Acad. Sci. USA.

[8] Tang L.J., Luo X.J., Tu H., Chen H., Xiong X.M., Li N.S., Peng J. (2021). Ferroptosis occurs in phase of reperfusion but not ischemia in rat heart following ischemia or ischemia/reperfusion. Naunyn. Schmiedebergs Arch. Pharmacol..

[9] Li J., Cao F., Yin H.-L., Huang Z.-J., Lin Z.-T., Mao N., Sun B., Wang G. (2020). Ferroptosis: Past, present and future. Cell Death Dis..

[10] Tang L.J., Zhou Y.J., Xiong X.M., Li N.S., Zhang J.J., Luo X.J., Peng J. (2021). Ubiquitin-specific protease 7 promotes ferroptosis via activation of the p53/TfR1 pathway in the rat hearts after ischemia/reperfusion. Free Radic. Biol. Med..

[11] Stamenkovic A., O’Hara K.A., Nelson D.C., Maddaford T.G., Edel A.L., Maddaford G., Dibrov E., Aghanoori M., Kirshenbaum L.A., Fernyhough P. (2021). Oxidized phosphatidylcholines trigger ferroptosis in cardiomyocytes during ischemia/reperfusion injury. Am. J. Physiol. Heart Circ. Physiol..

[12] Hanus J., Zhang H., Chen D.H., Zhou Q., Jin P., Liu Q., Wang S. (2015). Gossypol acetic acid prevents oxidative stress-induced retinal pigment epithelial necrosis by regulating the Fo_x_O_3_/Sestrin_2_ pathway. Mol. Cell Biol..

[13] El-Sharaky A.S., Newairy A.A., Elguindy N.M., Elwafa A.A. (2010). Spermatotoxicity, biochemical changes and histological alteration induced by gossypol in testicular and hepatic tissues of male rats. Food Chem. Toxicol..

[14] El-Sharaky A.S., Wahby M.M., Bader El-Dein M.M., Fawzy R.A., El-Shahawy I.N. (2009). Mutual anti-oxidative effect of gossypol acetic acid and gossypol-iron complex on hepatic lipid peroxidation in male rats. Food Chem. Toxicol..

[15] Reynolds J.M., Tone J.N. (1988). Subchronic oral administration of gossypol-acetic acid (GAA) alters the distribution and utilization of radioiron in male rats. Drug Chem. Toxicol..

[16] Lin J.H., Ting P.C., Lee W.S., Chiu H.W., Chien C.A., Liu C.H., Sun L.Y., Yang K.T. (2019). Palmitic acid methyl ester induces G(2)/M arrest in human bone marrow-derived mesenchymal stem cells via the p53/p21 pathway. Stem. Cells Int..

[17] Gadelha I.C., Fonseca N.B., Oloris S.C., Melo M.M., Soto-Blanco B. (2014). Gossypol toxicity from cottonseed products. Sci. World J..

[18] Haasler L., Kondadi A.K., Tsigaras T., von Montfort C., Graf P., Stahl W., Brenneisen P. (2021). The BH3 mimetic (±) gossypol induces ROS-independent apoptosis and mitochondrial dysfunction in human A375 melanoma cells in vitro. Arch. Toxicol..

[19] Cao H., Sethumadhavan K., Cao F., Wang T.T.Y. (2021). Gossypol decreased cell viability and down-regulated the expression of a number of genes in human colon cancer cells. Sci. Rep..

[20] He X., Wu C., Cui Y., Zhu H., Gao Z., Li B., Hua J., Zhao B. (2017). The aldehyde group of gossypol induces mitochondrial apoptosis via ROS-SIRT1-p53-PUMA pathway in male germline stem cell. Oncotarget.

[21] Shi X., Tao G., Ji L., Tian G. (2020). Sappanone a protects against myocardial ischemia reperfusion injury by modulation of Nrf2. Drug Des. Dev. Ther..

[22] Xu B., Zhang J., Strom J., Lee S., Chen Q.M. (2014). Myocardial ischemic reperfusion induces de novo Nrf2 protein translation. Biochim. Biophys. Acta.

[23] Shokeir A.A., Hussein A.M., Barakat N., Abdelaziz A., Elgarba M., Awadalla A. (2014). Activation of nuclear factor erythroid 2-related factor 2 (Nrf2) and Nrf-2-dependent genes by ischaemic pre-conditioning and post-conditioning: New adaptive endogenous protective responses against renal ischaemia/reperfusion injury. Acta Physiol..

[24] Keshmiri-Neghab H., Goliaei B. (2014). Therapeutic potential of gossypol: An overview. Pharm. Biol..

[25] Flak D.K., Adamski V., Nowaczyk G., Szutkowski K., Synowitz M., Jurga S., Held-Feindt J. (2020). AT101-loaded cubosomes as an alternative for improved glioblastoma therapy. Int. J. Nanomed..

[26] Xiang W., Yang C.Y., Bai L. (2018). MCL-1 inhibition in cancer treatment. Onco. Targets Ther..

[27] Fan Z., Cai L., Wang S., Wang J., Chen B. (2021). Baicalin prevents myocardial ischemia/reperfusion injury through inhibiting ACSL4 mediated ferroptosis. Front. Pharmacol..

[28] Li T., Tan Y., Ouyang S., He J., Liu L. (2022). Resveratrol protects against myocardial ischemia-reperfusion injury via attenuating ferroptosis. Gene.

[29] Yu Z.P., Yu H.Q., Li J., Li C., Hua X., Sheng X.S. (2020). Troxerutin attenuates oxygen-glucose deprivation and reoxygena-tion-induced oxidative stress and inflammation by enhancing the PI3K/AKT/HIF-1α signaling pathway in H9C2 cardiomyocytes. Mol. Med. Rep..

[30] Zhu X., Zhao Y., Hou W., Guo L. (2019). MiR-153 regulates cardiomyocyte apoptosis by targeting Nrf2/HO-1 signaling. Chromo-Some Res..

[31] Kasai S., Mimura J., Ozaki T., Itoh K. (2018). Emerging regulatory role of Nrf2 in iron, heme, and hemoglobin metabolism in physiology and disease. Front. Vet. Sci..

[32] Masini E., Vannacci A., Marzocca C., Pierpaoli S., Giannini L., Fantappié O., Mazzanti R., Mannaioni P.F. (2003). Heme oxygenase-1 and the ischemia-reperfusion injury in the rat heart. Exp. Biol. Med..

[33] Chiang S.K., Chen S.E., Chang L.C. (2018). A dual role of heme oxygenase-1 in cancer cells. Int. J. Mol. Sci..

