# Supplementary material for: Gossypol Acetic Acid Attenuates Cardiac Ischemia/Reperfusion Injury in Rats via an Antiferroptotic Mechanism
Source: Biomolecules. 2021 Nov 10;11(11):1667. doi: 10.3390/biom11111667 (PMC8615989; doi:10.3390/biom11111667)
Supplement: Supplementary file 1 [file biomolecules-11-01667-s001.zip › biomolecules-1408228-supplementary.pdf]

Supplement S1

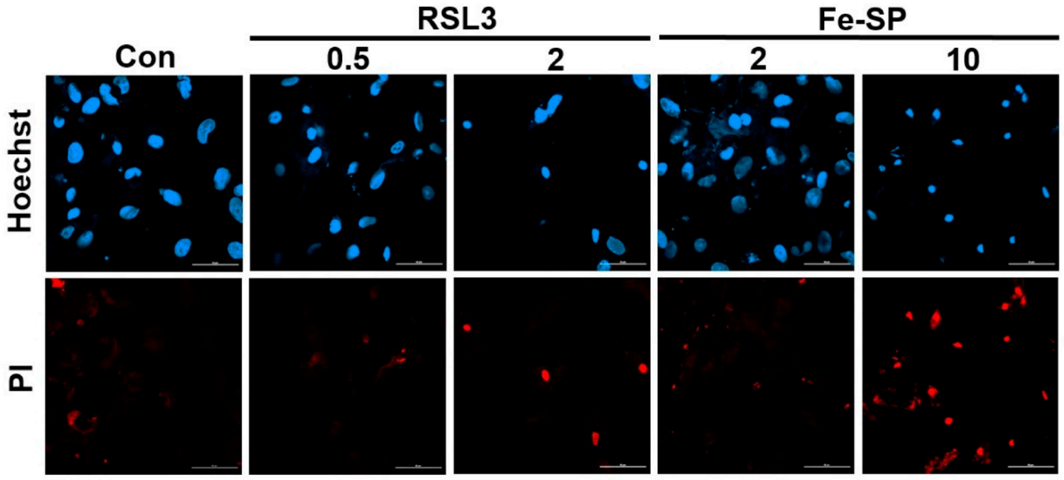

Figure S1. Treatment with RSL3 and Fe-SP in neonatal rat cardiomyocyte for 24 h, and examined though confocal microscopy using Hoechst 33342 and PI stainings. The results showed that RSL3 (2 μM) and Fe-SP (10 μM) treatments increased the number of PI positive cells, but not at low concentrations of RSL3 (0.5 μM) and Fe-SP (2 μM) in neonatal rat cardiomyocytes.
